# Effect of Supplementation of Vitamin A on Growth, Haemato-Biochemical Composition, and Antioxidant Ability in *Cyprinus carpio* var. *communis*

**DOI:** 10.1155/2022/8446092

**Published:** 2022-09-14

**Authors:** Aamina Hassan, Imtiaz Ahmed, Gohar Bilal Wani

**Affiliations:** ^1^Fish Nutrition Research Laboratory, Department of Zoology, University of Kashmir, Hazratbal, 190006, Srinagar, Jammu and Kashmir, India; ^2^Faculty of Fisheries, Sher-e-Kashmir University of Agricultural Sciences and Technology, Rangil, 191201, Ganderbal, Jammu and Kashmir, India

## Abstract

Vitamin A requirement in fingerling common carp, *Cyprinus carpio* var. *communis* (1.64 ± 0.02 g; ABW ± SD), was evaluated by conducting a 10 week growth experiment. Casein-gelatin-based test diets representing six graded levels of vitamin A (0, 0.03, 0.07, 0.11, 0.15, and 0.19 g/kg, dry diet) were designed and fed to the triplicate group of fish at 08:00 and 16:00 hrs at the rate of 4% body weight per day. Growth parameters like live weight gain (LWG %), feed conversion ratio (FCR), protein efficiency ratio (PER), specific growth rate (SGR), and body protein deposition (BPD) improved significantly (*P* < 0.05) with each elevated dietary vitamin A level and found maximum growth rate along with the best- FCR at 0.11 g/kg diet. Dietary vitamin A levels also significantly (*P* < 0.05) affected haematological parameters of the fish. Highest haemoglobin (Hb), erythrocyte count (RBC), haematocrit content (Hct %), and lowest leucocyte count (WBC) were observed at 0.11 g/kg vitamin A fed diet compared to all the diets. Highest protein and lowest fat content were observed in the group of fingerlings fed with 0.11 g/kg vitamin A containing diet. Blood and serum profile also showed some significant (*P* < 0.05) differences with elevating concentration of dietary vitamin A levels. Serum parameters like aspartate aminotransferase (AST), alanine aminotransferase (ALT), and cholesterol values decreased significantly (*P* < 0.05) at 0.11 g/kg vitamin A fed diet compared to control diet. However, except albumin the other electrolytes improved significantly (*P* < 0.05) and maximal values of these parameters were also evident at 0.11 g/kg of vitamin A fed diet. Better value of TBARS was found in the group that fed 0.11 g/kg vitamin A diet. Hepatosomatic index and condition factor improved significantly (*P* < 0.05) with fish fed at optimal dose 0.11 g/kg of vitamin A diet. Based on quadratic regression analysis of LWG%, FCR, BPD, Hb, and calcium values of *C. carpio* var. *communis* against the varying levels of dietary vitamin A, an optimum growth, best FCR, higher BPD, Hb, and Ca values lie in the range of 0.10 to 0.12 g/kg diet, respectively. The data generated during this study would be important in developing vitamin A balanced feed for successful intensive culture of *C. carpio* var. *communis.*

## 1. Introduction

Species compliable, nutritionally balanced formulated feed is prerequisite for profitable aquaculture. The development of such type of feed depends on complete information regarding dietary nutritional requirements and their respective availability in the feed ingredients [1–3]. Among all the essential nutrients supplemented through the artificial feed for growth, survival and maintenance of animals including fish,vitamins are figured as most vital micro nutrients [4]. They belong to the organic compounds which carry out specific functions in the body of fish and are needed in small quantities, obtained through an external source (diet) for health, growth and development, and reproduction [5–7]. Vitamins play a vital role in the process of metabolism by acting as cofactors to several enzymes during metabolic pathways. Therefore, health status and homeostasis of fish may be affected by taking inadequate amount of any vitamin [8].

Among the fat-soluble vitamins, first fat-soluble vitamin to be recognized is vitamin A, which is considered a vital micronutrient required for growth of fish and exists in three forms, namely, retinal, retinol, and retinyl esters [9, 10]. In fish, vitamin A plays a crucial role in vision, immunity, growth, oxidation resistance, glucose and lipid metabolism, erythropoiesis, and in the regulation of iron metabolism. Furthermore, it is directly or indirectly concerned in the activation of transcription factors of more than 500 genes in fish [6, 11–13]. Vitamin A acts as steroid hormone responsible for regulating growth through glycoprotein and glycosaminoglycan synthesis which results in differentiation of epithelial tissues and is thus considered one of the key physiological functions of vitamin A. Apart from playing part in such diverse functions, it certainly is one of the most versatile vitamins. Since synthesis of vitamin A in fish is absent, hence it must be supplied through diet [8, 14].

Nutritional requirement experiments of vitamin A have been conducted on several fish species [6], common carp, *C. carpio communis* [15], Atlantic salmon, *Salmo salar* [16], Nile mouth breeder, *Oreochromis niloticus* [17], senegalese sole, *Solea senegalensis* [18], wuchang bream, *Megalobrama amblycephala* [19], gibel carp, *Carassius auratus* var.gibelio [20], grass carp, *Ctenopharyngodon idella* [21, 22], orange spotted grouper, *Epinephelus coioides* [23], bagre, *Rhamdia quelen* [10], dorado, *Salminus brasiliensis* [24], northern whitening, *Silago sihama* [25], red bellied pacu, *Piaractus brachypomus* [26], black rock cod, *E. fuscoguttatus* and gaint grouper, *E. lanceolatus* [27], and Nile tilapia, *O. niloticus* [28]. Although dietary requirement of vitamin A in *C. carpio* var. *communis* has been determined before [15, 29], these authors have not mentioned the effect of vitamin A on haematology and serum biochemical parameters as used in the present study. Therefore, an attempt has been made to establish the vitamin A requirement of *C. carpio* var. *communis* in the present study by analyzing various growth as well as hemato-biochemical parameters.

*C. carpio* var. *communis* belongs to the largest fresh water family Cyprinidae and is highly nutritious, tasty, easily digested, more affordable, and an accessible one. Due to its high growth and easy cultivation, the fish is regarded as one of the most commonly cultured freshwater fish species all over the world [30–32]. Although the importance of vitamin A for normal growth, haemato-biochemical parameters, and serum profile is well documented for higher vertebrates, but to our knowledge scattered information is available on aquatic species including fish particularly *C. carpio* var. *communis.* Therefore, the present study aims to evaluate the effect of vitamin A supplementation on growth and haemato-biochemical parameters of *Cyprinus carpio* var. *communis* in order to establish information of vitamin A requirement of this species, which would be useful for the development of nutritionally balanced diet for optimal production of this fish species.

## 2. Materials and Methods

### 2.1. Formulation of Experimental Diets

Basal diet formulation for conducting this experiment is shown in Table 1. Six vitamin A based trial diets with the same quantity of protein (iso-nitrogeneous, 428 g/kg, crude protein) and uniform amount of energy (iso-caloric, 367 kcal100/g, gross energy) were formulated with casein (vitamin free) and gelatin substituted as protein, dextrin as a carbohydrate, and corn and cod liver oil as lipid sources, respectively. Different quantities of vitamin A (0, 0.03, 0.07, 0.11, 0.15, and 0.19 g/kg diet) were supplemented as retinyl acetate in all the diets by replacing *α*-cellulose. Quantities of vitamin A in the above formulated diets were set based on the information available in the existing literature [6]. These diets were labelled as A_1_, A_2_, A_3_, A_4_, A_5_, and A_6_. The mixtures of minerals and vitamins composition were as per Halver [14]. For the preparation of the test diets, firstly, gelatin was dissolved in a measured quantity of water separately with constant heating and stirring which was followed by the adding up of casein at 80°C. After removing the mixer bowl from heating, it was connected to a Hobart electric mixer (Hobart Corp., Troy, OH, USA), followed by the addition of dextrin and other ingredients. As the temperature of the bowl decreases around 40°C, premixes of vitamin and oil were added to the lukewarm in a sequential manner while stirring continuously. Eventually, carboxymethyl cellulose (CMC) was further added in the contents, while the velocity of the mixer was increased gradually till the diet started to become firm in its texture. The final diet with the consistency of bread dough was packed in sealed polythene bags and then stored at -4°C for future use.

### 2.2. Feeding Trial

Fingerlings of *C. carpio* var. *communis* in good condition were obtained from the Union Teritory, J and K, Government Fishery Department Manasbal Carp Hatchery. These fingerlings were transported to fish feeding trial laboratory at the Department of Zoology, University of Kashmir, Srinagar, India, in oxygen-filled transparent polythene bags. Fingerlings at first on arrival were given a prophylactic dip of KMnO_4_ (1 : 3000) about thirty seconds for the purpose of disinfection if any. These fingerlings were afterwards shifted to aqua blue coloured indoor circular plastic fish tanks (water volume capacity =60 L) for two weeks for acclimatization purpose. Throughout this time duration, fingerlings were given practical feed twice a day which was followed by their acclimatization for the next two weeks on casein- and gelatin-based vitamin A-free diet H-440 [14] to drain the body stores of vitamin A if any.

The fingerlings were sorted out from the above acclimatized lot and the required integer of *C. carpio* var. *communis* fingerlings with approximately same body mass (1.64 ± 0.15 g; ABW ± SD) was randamlydistributed in group of trio in 70 L circular polyvinyl tanks (water volume 60 L) fitted with uninterrupted water flow through system with 20 fish per tank at each level of dietary treatment. In each tank rate of water exchange was fixed at 1.0-1.5 L/min. As per the outcome in the feeding trial experiment conducted earlier in our laboratory, the moist cake form of test diet at the rate of 4% of the body weight was given to the experimental fish. The diets were fed at 08:00 and 16:00 hours by sub-dividing the ration into two equal portions. Feed was not given to fish on the day when weekly weight was to be recorded. Both initial as well as weekly weight was measured on a top-loading balance (0.1 mg, sensitivity, Sartorius CPA-224S, Goettingen, Germany) after anaesthetizing in tricaine methanesulphonate (100 *μ*g/ml). The experiment was terminated after lasted up to 10 weeks. The excreta of the fish were siphoned out before feeding on daily basis in every morning. Also, unutilized feed, if found, was collected back, dried, and weighed again to calculate the actual quantity of feed the fish consumed.

### 2.3. Carcass Analysis

Prior to the experiment, 40 fish species were sacrificed in a pool for initial proximate analysis and somatic indices studies. After recording the final weight on the last day of termination of experiment from each replicate, twelve fish were selected for the analysis of final proximate composition. Standard methods of AOAC [33] were employed to determine the proximate composition of test diets, initial and final body composition. Samples were oven dried at 105 ± 1°C for 22 h to estimate dry matter, while crude protein content of each sample was estimated using Kjeltec 8400 (FOSS Denmark) based on the Kjeldhal method. Estimation of crude lipid was done using solvent extraction method (FOSS Avanti automatic 2050, Sweden) and ash content was determined by oven incineration at a temperature of 650°C for 2–4 h (Muffle furnace YSPL-532, India).

### 2.4. Blood Collection and Analysis

On the completion of feeding experiment, for analyzing haematological parameters, blood samples of five anaesthetized fish specimen were selected at random from each replicate of the different diet treatments from caudal vein and divided equally into two sets of heparinized and non-heparinized tubes. Blood in heparinized tubes was meant for the evaluation of Hb, RBCs, WBCs counts, and Hct % content by following the earlier adopted method [34] and blood collected in non-heparinized tubes was then quickly centrifuged in micro-centrifuge (REMI-12C) 4100 × g for 10 min at 4°C for serum collection. The serum was then separated and analyzed for serum parameters. Fixed volume (100 *μ*L) pipette was used to dispense serum samples on the reagent rotors. The samples for each dose were analyzed in triplicates for the analysis of parameters like ALT, AST, glucose, total protein, albumin, globulin, cholesterol, phosphorous, calcium, and potassium. The serum analytes were assessed using an automatic vet scan biochemistry analyzer VS2 (Abaxis, USA). Before collecting blood, body weight and length of fish were taken for calculating condition factor (K). The liver was also removed carefully by dissecting the same fish for calculating hepatosomatic index [35]. Remaining fish samples were taken for the analysis of thiobarbituric acid reactive substances (TBARS) assessed by following the protocol adopted by [34] and were measured as nmol MDA/g liver tissue.

### 2.5. Water Quality Analysis

The examination of water quality was done on alternative day basis by recording some physio-chemical parameters like temperature, free carbon dioxide, dissolved oxygen, total alkalinity, and pH of the experimental water as per the standard methods of APHA [36]. In early morning before feeding the fish, water sample was collected for analysis. Water temperature noted with the help of mercury thermometer was found in the range of 23.2-24.8°C, and dissolved oxygen was estimated by Winkler's iodimetric test and was noted in the range of 6.2-7.4 mg/L. Similarly, free carbon dioxide and total alkalinity were evaluated by titrimetric methods and were found to be in the range of 4.2-6.6 mg/L and 92-120 mg/L, respectively, while pH 7.2-7.5 was measured by using a digital pH meter (pH ep-HI 98107, USA).

### 2.6. Statistical Analysis

After completion of the experiment, data obtained were analyzed by one-way analysis of variance (ANOVA) [37, 38]. Tukey's test (*P* < 0.05) was used to compare the mean values of replicates. To predict more accurate responses, the optimum dietary vitamin A level was determined by using second-degree polynomial regression (*Y* = *a* + *bx* + *cx*^2^) analysis [39]. Statistical analysis was made by means of Origin software (version 8.5.1; San Clemente, CA.

## 3. Results

Growth parameters of fingerling *C. carpio* var. *communis* fed varying doses of dietary vitamin A are listed in Table 2, which clearly indicates that LWG%, SGR, FCR, PER, and BDP attributes of fish which was fed the diets possessing rising levels of vitamin A varied significantly (*P* < 0.05). Fish fed control diet showed poor growth in terms of LWG%, SGR, PER, and BPD whereas those fed 0.11 g/kg vitamin A diet exhibited maximum LWG (387%), SGR (2.26), PER (1.51), and BPD (27.31) followed by decrease in all these parameters that was noted with respect to further increase of vitamin A in the diet. The FCR followed decreasing trend up to the dose 0.11 g/kg vitamin A level; after that, it starts to increase again in doses containing higher concentration of vitamin A. The survival rate (SR) improved significantly (*P* < 0.05) in supplemented groups in comparison to the control group.

Whole body composition of the experimental fish and somatic indices are listed in Table 3. Dietary vitamin A levels significantly (*P* < 0.05) influenced the moisture, crude protein, fat, and ash contents of all groups. Crude protein showed a positive rising trend as dietary vitamin A level increased in the diet up to 0.11 g/kg, after that the values of protein decrease.The body fat content decrease with each incremental level and lowest fat content was recorded in fish fed diet containing 0.11 g/kg vitamin A supplied diet beyond that it starts to rise again. Moisture increased significantly (*P* < 0.05) up to 0.11 g/kg diet of vitamin A beyond which it remains almost constant, while whole body ash shows insignificant difference (*P* > 0.05) with respect to each feeding level. Further, our study reveals that feeding different levels of dietary vitamin A to fingerlings significantly affected (*P* < 0.05) somatic indices. Highest hepatosomatic index (HSI) 1.72 and lowest condition factor (K) 1.28 were recorded in fish fed control diet, which later onwards improved significantly (*P* < 0.05) with the incremental levels of dietary vitamin A up to 0.11 g/kg of dry diet beyond which no improvement was apparent, signifying the good condition of fish in the group fed optimal dose of 0.11 g/kg level of dietary vitamin A.

Haematological parameters of fish fed varying levels of dietary vitamin A are depicted in Table 4. Dietary vitamin A supplementation significantly (*P* < 0.05) affected red blood cells (RBCs), white blood cells (WBCs), haemoglobin (Hb), and hematocrit (Hct) concentration. Highest values for Hb (9.78 g/dl), RBCs (3.91×106 mm^−3^), and Hct (30.89%) were observed for the group fed diet containing 0.11 g/kg vitamin A diet beyond which no significant (*P* < 0.05) differences were recorded in fish fed diets containing elevated concentration of vitamin A. Contrary to this, lowest value of WBCs (2.27×106 mm^−3^) was recorded in the group fed 0.11 g/kg diet of vitamin A. However, TBARS activity improves with fish fed different levels of dietary vitamin A diets and minimum TBARS value was also achieved in fish fed 0.11 g/kg of vitamin A. It was also observed that value of TBARS was minimum in fish fed higher levels of vitamin A and maximum in the control group.

Serum biochemical parameters of the present study also showed significant (*P* < 0.05) variation with respect to each incremental level and the results are presented in Table 5. Serum cholesterol, ALT, and AST decreased significantly (*P* < 0.05) up to the group of fingerlings fed 0.11 g/kg vitamin A diet and thereafter, it started to increase as the concentration of vitamin A in diet is elevated. Contrary to this, serum glucose, globulin phosphorous, calcium, and potassium values increased significantly (*P* < 0.05) up to the optimal dose of 0.11 g/kg level of vitamin A diet and thereafter, a decrease in these parameters was obtained with further incremental levels of dietary vitamin A. However albumin and total protein contents do not show any significant (*P* > 0.05) variation, when fed varied levels of vitamin A in the study.

**Figure 1:**
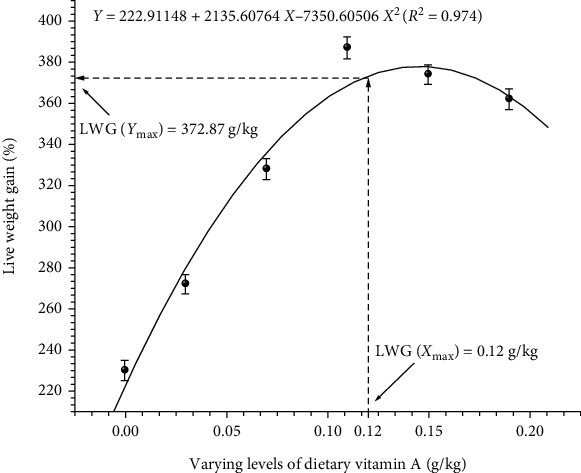
Quadratic regression analysis of dietary vitamin A levels to live weight gain. Each point represents the mean of three replicates per treatment.

**Figure 2:**
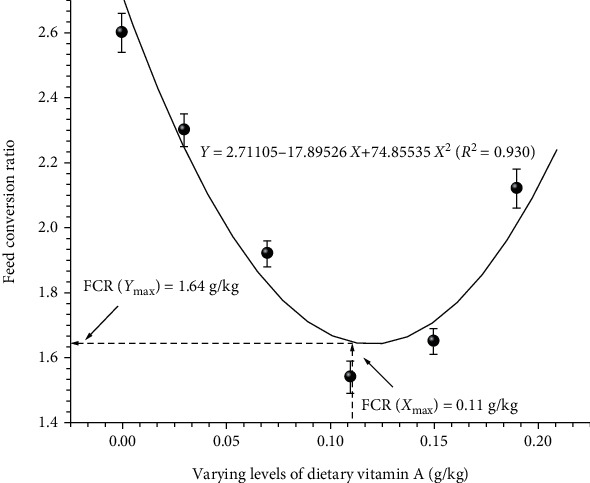
Quadratic regression analysis of dietary vitamin A levels to feed conversion ratio. Each point represents the mean of three replicates per treatment.

**Figure 3:**
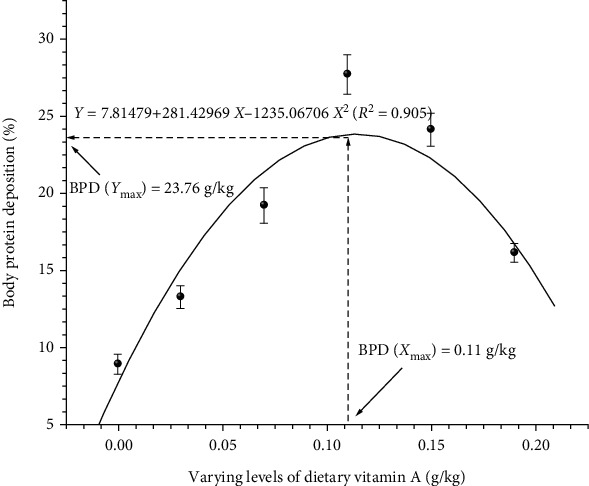
Quadratic regression analysis of dietary vitamin A levels to body protein deposition. Each point represents the mean of three replicates per treatment.

**Figure 4:**
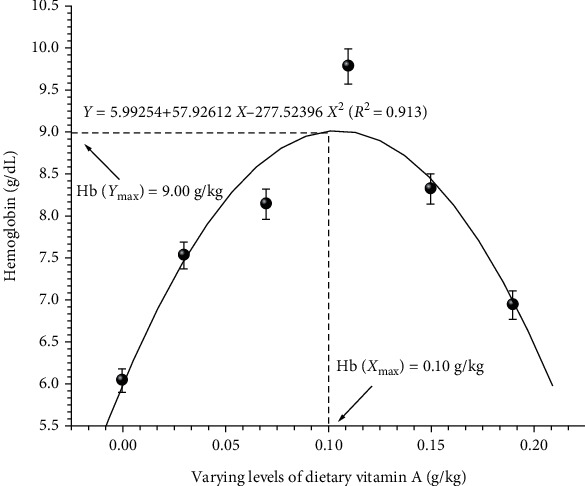
Quadratic regression analysis of dietary vitamin A levels to haemoglobin. Each point represents the mean of three replicates per treatment.

**Figure 5:**
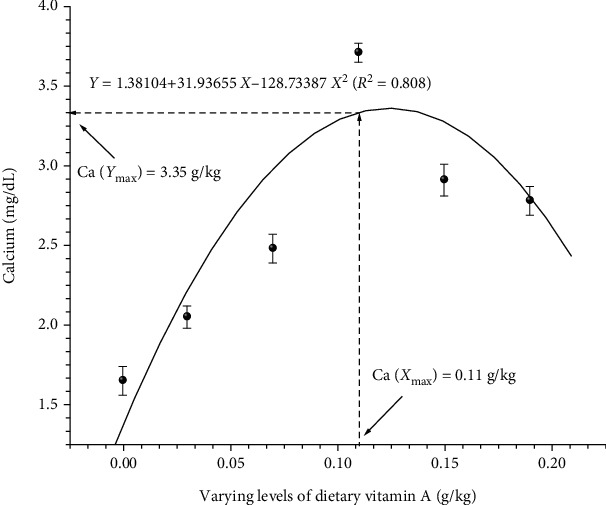
Quadratic regression analysis of dietary vitamin A levels to calcium. Each point represents the mean of three replicates per treatment.

To obtain accurate information on vitamin A requirement of fish under study, data were subjected to quadratic regression analysis. Regression analysis of LWG% (Figure 1), FCR (Figure 2), BPD (Figure 3), Hb (Figure 4), and Ca (Figure 5) against varying levels of dietary vitamin A concentrations exhibited the requirement in the range of 0.10 to 0.12 g/kg of the diet, respectively.

## 4. Discussion

Species living in water must contend to a range of environmental factors including temperature, pressure, salinity, and oxygen availability as well as pollution concentration that influence nutrient requirements; hence, there occurs variation from those species living on land [40–43]. Unlike vitamins soluble in water, the liver acts as a storage organ for the fat-soluble vitamins. This storage of lipid-soluble vitamin in the liver can cause hypo or hypervitaminosis, that may lead to problems to the homeostasis of animals including fish [26], and hence, it becomes necessary to find an optimal requirement of fat-soluble vitamins. Vitamin A dietary requirement has been a hot topic in research field and it had been studied extensively. Vitamin A plays a pivotal role in supporting growth, histogenesis, nervous and skeleton system, morphogenesis, cell proliferation, and differentiation system development, in the life of early stages of vertebrate [13, 44–46]. Dietary vitamin A is also considered vital for growth of *C. carpio* var. *communis* as indicated in our study and its essentiality in fish has also been proved by previous studies as well [10, 13, 21, 22, 47–49]. Quadratic regression analysis of the LWG% data of this fish showed the optimal vitamin A requirement to be 0.11 g/kg diet. This vitamin A requirement of *C. carpio* var. *communis* is higher than reported for European seabass, *Dicentrachus labrax* [0.031 g/kg, [50]], *M. amblycephala* [0.001 g/kg, 19], O. *niloticus* [0.0012 g/kg, 8], *S. brasiliensis* [0.002 g/kg, 24], hybrid grouper, *E. fuscoguttatus × E. Lanceolatus* [0.089 g/kg, 27], *O*. *niloticus* [0.006 g/kg, 28], but lower than *C. carpio* var. *communis* [0.4 g/kg, 15]. The difference in the vitamin A requirements may be due to varying methodological approaches and assessment criteria used such as species and size of fish, feeding environment, and measures of management.

For the assessment of the overall health, nutritional status, and balance in water of various fish species, haematological parameters act as a main diagnostic tool in aquaculture [3]. Fish haematological studies are gaining momentum due to their role in understanding nutrient dynamics and impact of diets on the fish health [51–56]. It has been identified that vitamin A plays an important role in haematology. Donoghue et al. [57] and Guimaraes et al. [17] reported that haematological parameters might be better indicators of vitamin A deficiency as compared to growth. In the findings of our study, haematological parameters improved with elevating dietary vitamin A levels up to 0.11 g/kg. However, there was reduced Hb, Hct, and RBC count found in the fish offered control diet as compared to other group supplemented increased levels of dietary vitamin A. Same results were found in the studies of *C. carpio* var. *jain*, Yang et al. [58] and *O. niloticus*, Guimaraes et al. [17]. This might be due to the reason of reduced feed intake or absence of vitamin A in diet as this vitamin plays a great role in the haematopoietic function of fishes [8]. Another function of vitamin A is that it has got higher oxidation potential against oxidative stress in living body that damages proteins and lipids. During electron transfer in oxidation reactions of several metabolic processes, there occurs an imbalance in redox reactions, results in reactive oxygen species (ROS) production, hence causes oxidative stress [59–61]. Quantification of TBARS was done to take the measurements of oxidative damage [62, 63]. In our study, activity of TBARS is found maximum in control diet but slows down as the concentration of vitamin A in basal diet is elevated which agrees with the study of Battisti et al. [10] and Wu et al. [22].

The physiological and pathological changes in fish happened due to many factors both exogenous and endogenous, affected by balanced feed and temperature, as evident in serum biochemical parameters of fish measuring health, nutritional status, and the ability to adapt to environmental changes [64]. Stress could be a cause for change in fish serum biochemical indices as suggested by some researchers [65]. The detection of enzymes AST and ALT in the serum are most times evaluated in fish exposed to disease or malnutrition, stress, and toxins, as they indicate harm to the cells of the liver mainly hepatocytes and parenchymal cells [66, 67]. In our study, high ALT and AST activities were seen in the group of fish given lowest and highest concentration of vitamin A. This suggests damage to the liver of *C. carpio* var. *communis* occurred due to inadequate or excessive supplementation of vitamin A in the diet. Same outcome was reported by the study of *C. auratus* [20], *M. amblycephala* [19], Japanese flounder, *Paralichthys olivaceus* [68], and *C. idellus* [69]. Serum protein and albumin are also used for the evaluations of the liver condition and their increase shows strong immune response in fish [70, 71]. In fish, the measurements of total protein, albumin, and globulin in serum are of significant diagnostic value as it indicates general nutritional status, integrity of the vascular system, and better liver functioning [72]. In our experiment, although values of total protein and albumin do not vary significantly, but slight increase was found up to the diet containing 0.11 g/kg which then afterwards started decreasing. The declining serum albumin level in the present study might be due to lower feed intake, kidney and intestinal loss, and disturbed liver metabolism [73]. Electrolytes like potassium, phosphorous, and calcium have multiple physiological roles in the body like it controls osmotic equilibrium and cellular ionic exchange and maintains membrane permeability as well. The higher values of these parameters indicate higher passive permeability, depolarization, and destabilization of the membrane [74]. Glucose represents an immediate source of energy necessary for the function of the heart and muscles of fish body. Glucose values in our study showed an increasing pattern with increasing concentration of diet rich in vitamin A up to a dose containing 0.11 g/kg but after that it also started to decrease again in further doses with higher quantity of vitamin A. There is a direct correlation between blood glucose levels and environmental stress that in turn is reflected in carbohydrate metabolism of the fish [75].

In the present study, feeding varying levels of dietary vitamin A to *C. carpio* var. *communis* showed significant impact on proximate composition. Moisture, crude protein, and fat contents varied significantly. It is clearly visible in the results that fish fed basal diet showed highest fat content and lowest protein content, when compared to other group of fishes fed elevated levels of dietary vitamin A; the reason might be that fish fed basal diet preferred protein as an energy source than fat. Decreasing pattern of body fat concentration in fish fed diet containing higher levels of vitamin A than fish fed basal diet has also been reported in the past by other workers like Ornsrud et al. [76], Mohamed et al. [77], Hernandez et al. [78], Battisti et al. [10] and Wu et al. [22]. Also results obtained in fingerlings of *C. carpio*, Jeyaraj et al. [15], *E. coioides*, Yang et al. [23], and sturgeon *A. Schrenckii*, Wen et al. [46] showed significant difference in proximate composition in fishes fed varying levels of dietary vitamin A. However contradictory results were also reported in *C. idellus* [21].

The liver is a major storage organ involved with vitamin A metabolism and homeostasis [26]. In fish, at the time of high energy consumption, liver and muscle tissues act as an organ of storage for energy in the form of fat and glycogen that leads to enlargement of the liver. The determination of this enlarged liver indicates health status of the fish [34]. In this experiment, higher hepatosomatic index (HSI) was found in fish given control diet and was decreased as concentration of dietary vitamin A in diet rises which is in agreement with other studies conducted on sunshine bass, *Morone chrysops× M. saxatilis*, Hemre et al. [79], *Salmo salar*, Ornsrud et al. [16], *M. amblycephala*, Liu et al. [19], *E. coioides*, Yang et al. [23], and *Mycteroperca tigris × E. lanceolatus*, Liang et al. [27]. The reason behind this could be that fish prefer to utilize protein as an energy source over other nutrients like carbohydrates and lipids which results in deposition of fat in the liver of fish that in turn shows higher value of HSI. Another reason for liver enlargement would be deficiency of vitamin A that leads to fat accumulation [27, 80]. As per Tripatti et al. [81], enhanced lipid droplets and lesions occur because of infiltration of inflammatory cells in the liver when dietary vitamin A is consumed in excess. It also promotes fatty acid decomposition by promoting the coding of the mitochondria while enhancing expression of fatty acid oxidase gene that results in liver enlargement. On the other hand, next significant tool that is often used to measure the nutritional condition of fish and health as well is condition factor (K). It provides information of proper utilization of feed given to the fish [82, 83]. In our research, lowest condition factor was found in control group which resembles with the study of *A. Schrenckii*, Wen et al. [46] and highest CF was found in fish fed 0.11 g/kg vitamin A which indicated that this dietary vitamin A dose was better for optimal growth of fish under study, which is in agreement with the study on *R. quelen*, Battisti et al. [10] and *M. tigris × E. lanceolatus*, Liang et al. [27] when fed vitamin A diet to these fishes.

Vitamin A is among those nutrients which when absent or superfluous can do damage to the biological health [25]. Elimination of vitamin A in fish diet can cause many signs of deficiency such as greater mortality, retarded growth, poor feed efficiency, anaemia, haemorrhages on the head, fin erosion, erratic swimming, exophthalmia, cataracts, lethargy, and loss of scales [6, 14, 24, 58, 77, 84] In addition, vitamin A nutrient helps in the maintenance of structure as well as function of the digestive tract of juvenile Jain carp as reported by Uni et al. [85] and Yang et al. [58]; they also added that its deficiency decreased intestinal fold also in the jain carp. Also, necropsies of dead fish were done as opportune to verify internal deficiency signs like hemorrhagic kidney, spleen, and pale liver, but no such type of deficiency signs was found in our experiment except poor growth, poor feed conversion efficiency, and lower haematological indices. Similarly, Liu et al. [19] have reported no signs of vitamin A deficiency except poor growth, protein efficiency ratio, and feed consumption in *M. amblycephala*; contrary to this, Wen et al. [46] reported that the absence of vitamin A deficiency in *A. schrenckii* exhibited poor appetite. Hernandez et al. [69] also reported feed avoidance and retarded growth as deficiency signs in *P. olivaceus* fed with basal diet without supplemental vitamin A. Haung et al. [25] reported greater mortality, erosion, haemorrhages, and sluggish movement in *S. Sihama*, when fed control diet and poor appetite when fed with diet containing higher concentration of dietary vitamin A.

## 5. Conclusion

The results of the study shows the importance of dietary vitamin A inclusion for fingerling *C. carpio* var. *communis* to attain maximal growth and best feed conversion. Proximate composition, antioxidant capacity, haematology, and serum profile of *C. carpio* var. *communis* improved significantly with the optimum inclusion of vitamin A. Based on quadratic regression analysis of LWG, FCR, BPD, Hb, and Ca data against varying levels of dietary vitamin A, 0.11 g/kg vitamin A is optimal for growth of fingerling *C. carpio* var. *communis*. The data generated during this study would be important in developing vitamin A balanced feed for successful intensive culture of *C. carpio* var. *communis.*

## Figures and Tables

**Table 1:** Composition of the basal diet used for estimating the dietary vitamin A requirement of *Cyprinus carpio* var. *communis* fingerling.

Ingredients	Amount (g/kg dry diet)
Casein^∗^	414.6
Gelatin^‡^	103.6
Dextrin	279.7
Corn oil	60.0
Cod liver oil	30.0
Mineral mix^†^	40.0
Vitamin mix (a-free)^†,§^	9.61
Carboxymethyl cellulose	40.0
Alpha cellulose	22.29
Tetracycline	0.20
Total	1000
Proximate analysis (*N* =3)	
Analyzed crude protein (g/kg)	427.70
Fat (g/kg)	89.65
Moisture	88.50
Gross energy*^Ψ^*(Kcl 100/g, dry diet)	367.0

^∗^Crude protein (80%); ^‡^crude protein (93%), Loba Chemie, India; ^†^Halver,2002 mineral (AlCl3.6H2O, 15 mg; ZnSO4.7H2O, 300 mg; CuCl, 10 mg; MnSO4.4-6H2O, 80 mg; KI, 15 mg; CoCl2.6H2O, 100 mg; plusUSP#2Ca(H2PO4)2.H2O, 13.58 g; C6H10CaO6, 32.70 g; C6H5O7Fe.5H2O, 2.98 g; MgSO4.7H2O, 13.20 g; KH2PO4 (dibasic), 23.98 g; NaH2PO4.2H2O, 8.72 g; NaCl, 4.35 g (g100/g); ^†,§^vitamin mix (vitamin A variable; choline chloride, 500 mg: thiamin, 5.0 mg; riboflavin, 20.0 mg; calcium pentothenate, 50.0 mg; inositol, 200.0 mg; biotin, 0.50 mg; folic acid, 1.50 mg; ascorbic acid, 100.0 mg; menadione, 4.0 mg; alpha-tocophery acetate, 40.0 mg; cyanocobalamine, 0.01 mg 100/g diet. ^Ψ^Calculated on the basis of fuel values 18.82, 14.64, and 35.56 kJ g^−1^ for protein, carbohydrate and fat, respectively, as estimated on Gallenkamp ballistic bomb calorimeter [86].

**Table 2:** Growth performance of fingerling *Cyprinus carpio* var. *communis* fed diets containing varying levels of vitamin A^a,b^.

Varying levels of vitamin A (g/kg dry diet)							
	0 (A1)	0.03 (A2)	0.07 (A3)	0.11 (A4)	0.15 (A5)	0.19 (A6)	*P* value
Average initial weight (g)	1.64 ± 0.02	1.62 ± 0.02	1.67 ± 0.03	1.64 ± 0.03	1.65 ± 0.02	1.68 ± 0.02	0.649
Average final weight (g)	5.41 ± 0.01^d^	6.02 ± 0.15^c^	7.14 ± 0.18^b^	8.03 ± 0.19^a^	7.82 ± 0.16^a^	7.76 ± 0.17^ab^	0.033
Live weight gain (%)^c^	230 ± 3.34^f^	272 ± 4.20^e^	328 ± 3.91^d^	387 ± 4.44^a^	374 ± 4.30^b^	362 ± 3.55^c^	0.025
Specific growth rate (%/day)^d^	1.70 ± 0.01^e^	1.87 ± 0.02^e^	2.07 ± 0.02^d^	2.26 ± 0.01^a^	2.22 ± 0.02^b^	2.18 ± 0.02^c^	0.025
Feed conversion ratio^e^	2.6 ± 0.06^a^	2.3 ± 0.05^b^	1.92 ± 0.04^d^	1.54 ± 0.05^f^	1.65 ± 0.04^e^	2.12 ± 0.06^c^	0.014
Protein efficiency ratio (%)^f^	0.89 ± 0.02^d^	1.01 ± 0.06^d^	1.21 ± 0.03^bc^	1.51 ± 0.05^a^	1.41 ± 0.06^b^	1.10 ± 0.03^c^	0.036
Body protein deposition (g/fish)^g^	10.39 ± 0.87^f^	13.63 ± 0.81^e^	18.77 ± 1.23^c^	27.31 ± 1.69^a^	22.87 ± 1.14^b^	15.76 ± 0.76^d^	0.497
Survival (%)	90	95	100	100	100	97	

^a^Mean values of 3 replicates ± SEM. ^b^Mean values sharing the same superscripts in the same row are not significantly different (*P* > 0.05). ^c^Live weight gain(%) = final body weight (g)–initial body weight (g)/initial body weight × 100. ^d^Specific growth rate = 100 × ln (mean final weight) − ln (mean initial weight)/no.of days. ^e^Feed conversion ratio (FCR) = feed given (dry weight basis)/wet weight gain (g). ^f^Protein efficiency ratio (PER) = wet weight gain (g)/protein consumed. ^g^Body protein deposition = 100 × (final body weight × final body protein)–(initial body weight × initial body protein)/total amount of diet consumed × percentage of crude protein in the diet.

**Table 3:** Body composition and somatic indices of fingerling *Cyprinus carpio* var. *communis* fed diets containing varying levels of vitamin A^a,b^.

Varying levels of vitamin A (g/kg dry diet)								
	Initial	0 (A 1)	0.03 (A 2)	0.07 (A 3)	0.11 (A 4)	0.15 (A 5)	0.19 (A6)	*P* value
Moisture (%)	79.95 ± 0.07	75.24 ± 0.05^d^	76.18 ± 0.06^c^	78.09 ± 0.09^b^	79.43 ± 0.07^a^	79.28 ± 0.05^a^	79.64 ± 0.07^a^	0.031
Protein (%)	12.62 ± 0.09	10.75 ± 0.05^f^	12.95 ± 0.06^e^	15.05 ± 0.08^c^	17.11 ± 0.09^a^	16.05 ± 0.04^b^	14.21 ± 0.05^d^	0.024
Fat (%)	3.41 ± 0.02	4.53 ± 0.03^a^	4.12 ± 0.04^b^	3.67 ± 0.03^c^	3.28 ± 0.04^d^	3.32 ± 0.02^d^	3.51 ± 0.04^cd^	0.035
Ash (%)	3.10 ± 0.01	2.92 ± 0.05^a^	2.82 ± 0.03^a^	2.74 ± 0.06^ab^	2.90 ± 0.04^a^	2.87 ± 0.03^a^	2.76 ± 0.05^a^	0.256
Hepatosomatic index (%)^c^	2.12 ± 0.06	1.72 ± 0.04^a^	1.07 ± 0.05^b^	0.78 ± 0.02^c^	0.59 ± 0.03^d^	0.65 ± 0.03^d^	0.71 ± 0.02^cd^	0.011
Condition factor (g/cm^3^)^d^	1.71 ± 0.02	1.28 ± 0.06^d^	1.45 ± 0.05^c^	1.71 ± 0.07^b^	1.91 ± 0.05^a^	1.86 ± 0.04^a^	1.89 ± 0.06^a^	0.031

^a^Mean values of 3 replicates ± SEM. ^b^Mean values sharing the same superscripts in the same row are not significantly different (*P* > 0.05). ^c^Hepatosomic index (%) = liver weight (g)/body weight(g) × 100. ^d^Condition factor = body weight (g)/body length (cm)^3^ × 100.

**Table 4:** Haematological activity of fingerling *Cyprinus carpio* var. *communis* fed diets containing varying levels of vitamin A^a,b^.

Varying levels of vitamin A (g/kg dry diet)							
	0 (A1)	0.03 (A2)	0.07 (A3)	0.11 (A4)	0.15 (A5)	0.19 (A6)	*P* value
Haemoglobin (g/dL)	6.04 ± 0.13^e^	7.53 ± 0.16^d^	8.14 ± 0.12^b^	9.78 ± 0.25^a^	8.32 ± 0.11^b^	6.94 ± 0.17^c^	0.036
Haematocrit value (HCT %)	18.51 ± 0.23^f^	23.37 ± 0.39^e^	29.72 ± 0.17^c^	35.89 ± 0.15^a^	32.17 ± 0.19^b^	26.47 ± 0.13^d^	0.039
RBC (× 10^6^/mm^3^)^c^	2.05 ± 0.13^d^	2.49 ± 0.15^c^	3.04 ± 0.17^b^	3.91 ± 0.15^a^	2.56 ± 0.18^c^	1.75 ± 0.17^e^	0.046
WBC (× 10^4^/mm^3^)^d^	2.71 ± 0.06^a^	2.53 ± 0.05^b^	2.41 ± 0.03^c^	2.27 ± 0.02^d^	2.35 ± 0.03^e^	2.43 ± 0.05^c^	0.038
TBARS (nmol MDA/g liver tissue)^e^	2.53 ± 0.05^a^	2.09 ± 0.04^b^	1.64 ± 0.03^c^	1.31 ± 0.04^d^	1.42 ± 0.02^cd^	1.58 ± 0.03^c^	0.012

^a^Mean values of 3 replicates ± SEM. ^b^Mean values sharing the same superscripts in the same row are not significantly different (*P* > 0.05). ^c^RBCs = red blood cell count. ^d^WBCs = white blood cell count. ^e^TBARS = thiobarbituric acid reactive substances.

**Table 5:** Serum biochemical profile of fingerling *Cyprinus carpio* var*. communis* fed diets containing varying levels of dietary vitamin A^a,b^.

Varying levels of vitamin A (g/kg dry diet)							
	0 (A1)	0.03 (A2)	0.07 (A3)	0.11 (A4)	0.15 (A5)	0.19 (A6)	*P* value
Glucose (mmol/L)	5.23 ± 0.04^e^	5.72 ± 0.05^d^	6.43 ± 0.04^c^	8.05 ± 0.06^a^	7.89 ± 0.05^b^	6.82 ± 0.05^c^	0.044
Albumin (g/L)	11.21 ± 0.81^a^	11.32 ± 0.76^a^	11.57 ± 0.91^a^	11.86 ± 0.78^a^	11.12 ± 0.69^a^	11.34 ± 0.83^a^	0.468
Globulin (g/L)	12.67 ± 0.38^c^	13.16 ± 0.54^b^	13.35 ± 0.93^b^	14.09 ± 0.86^a^	13.41 ± 0.72^b^	13.37 ± 0.67^b^	0.245
Total serum protein (g/dl)	23.81 ± 1.86^c^	24.50 ± 1.69^b^	24.90 ± 1.74^b^	25.92 ± 1.80^a^	24.52 ± 1.75^b^	24.71 ± 1.83^b^	0.447
Alanine aminotransferase (U/L)	47.37 ± 1.62^a^	32.45 ± 2.54^b^	24.28 ± 1.46^c^	14.13 ± 1.42^d^	22.68 ± 1.56^c^	23.52 ± 1.35^c^	0.014
Aspartate aminotransferase (U/L)	139.92 ± 3.58^a^	131.07 ± 2.47^b^	122.63 ± 2.31^c^	112.18 ± 2.25^d^	120.41 ± 3.47^c^	118.25 ± 2.61^c^	0.025
Cholesterol (mmol/L)	3.76 ± 0.04^a^	2.84 ± 0.06^b^	2.06 ± 0.05^c^	1.16 ± 0.07^d^	2.12 ± 0.04^c^	2.17 ± 0.06^c^	0.032
Phosphorus (mg/dL)	1.19 ± 0.05^f^	1.92 ± 0.05^e^	2.67 ± 0.08^b^	3.56 ± 0.07^a^	2.41 ± 0.04^c^	2.23 ± 0.06^d^	0.049
Calcium (mg/dL)	1.65 ± 0.02^f^	2.05 ± 0.04^e^	2.48 ± 0.03^d^	3.71 ± 0.05^a^	2.91 ± 0.06^b^	2.78 ± 0.03^c^	0.043
Potassium (mmol/L)	0.67 ± 0.01^f^	1.34 ± 0.03^de^	1.61 ± 0.04^c^	2.08 ± 0.05^a^	1.83 ± 0.02^b^	1.41 ± 0.05^d^	0.044

^a^Mean values of 3 replicates ± SEM. ^b^Mean values sharing the same superscripts in the same row are not significantly different (*P* > 0.05).

## Data Availability

The data used to support the findings of this study are available with the corresponding author.
